# Treatment of Ectopic Mandibular Second Permanent Molar with Elastic Separators

**DOI:** 10.1155/2014/621568

**Published:** 2014-06-22

**Authors:** R. Rajesh, V. Naveen, S. Amit, Kusai Baroudi, C. Sampath Reddy, Srinivas Namineni

**Affiliations:** ^1^Department of Paediatric and Preventive Dentistry, KLR'S Lenora Institute of Dental Sciences, Rajanagaram, Rajahmundry, Andhra Pradesh 533294, India; ^2^Department of Paediatric and Preventive Dentistry, Sibar Institute of Dental Sciences, Guntur, Andhra Pradesh 522 509, India; ^3^Department of Paediatric and Preventive Dentistry, S.V.S Institute of Dental Sciences, Mahabubnagar, Andhra Pradesh 509 001, India; ^4^Department of Restorative Dental Sciences, Al-Farabi College, Riyadh 11691, Saudi Arabia; ^5^Department of Paediatric and Preventive Dentistry, Sri Sai College of Dental Surgery, Kothrepally, Vikarabad, Andhra Pradesh 501101, India

## Abstract

Ectopic eruption is a developmental disturbance in which the tooth fails to follow its normal eruption pathway. Ectopic eruption of the second molar is relatively rare. This paper presents the case of thirteen-year-old male with an ectopic mandibular second permanent molar. The condition was corrected with surgical exposure and placement of elastic separators. This case report lays emphasis on the practice of basic methods to obtain acceptable results rather than extensive surgical or orthodontic corrections. It is advised that ectopic teeth should not be neglected especially when it concerns developing caries and malocclusion.

## 1. Introduction

Tooth eruption is a complex, localized, and programmed process involving the bone remodeling at a precise timing. Inclined mesially, tooth buds of permanent second molar develop distal to permanent first molar. Remodeling of mandibular ramus corrects this anomaly, failure of which may lead to malocclusion. One such malocclusion is ectopic eruption.

Ectopic eruption occurs due to the deviation in normal path of eruption path leading to tooth locked apical to the distal surface of the molar. Ectopic eruption is more in maxillary first permanent molars and canines, followed by the mandibular canine, mandibular second premolar, and the maxillary lateral incisors [1, 2]. Prevalence of ectopic eruption of the lower permanent second molar is reported between 0.06 and 0.3% [3–5]. According to Raghoebar, the primary cause of ectopic eruption of permanent second molar is arch length deficiency [6].

Impaction is one of the conditions which mimics ectopic eruption of the teeth. Impaction is the lack of eruption of a tooth caused by an obstruction clinically or radiographically detectable or due to an abnormal direction of the tooth [6].

Primary failure of eruption could be due to metabolic disturbance in the dental follicle; subsequently bone resorption fails to initiate [7]. Radiograph shows normal eruption pathway.

Ankylosis and submerged tooth are similar conditions, where in cessation of eruption of a tooth occurs after emergence [8]. In this condition neither physical barrier nor abnormal position could be detected.

Ectopic eruption can be diagnosed clinically and radiographically (IOPA). Failure to treat ectopic second molar at the right time may lead to resorption of first permanent molar, caries, and subsequently elicit pain. Ideal period to treat ectopic mandibular second molars is from 11 to 14 years of age with incomplete root formation. Various factors influencing the treatment options are inclination of the tooth and depth of the second molar with reference from the first permanent molar.

The goals of the treatment are space regaining, up righting of the molar, and establishment of the normal occlusion.

This paper presents a case report with unilateral ectopic mandibular second molar treated with elastic separators.

## 2. Case Description

A 13-year-old male reported to the Department of Paediatric and Preventive Dentistry with a chief complaint of dental pain in right mandibular posterior region. Pain was mild continuous, vague aching pain. Patient presented no significant medical history and history of trauma. On extra oral examination, the patient was found to have a straight profile and a symmetric face. Clinical intraoral examinations revealed good oral hygiene with arrested caries in the right first permanent molar. The molar relationship was Angle class I on both sides.

**Figure 1:**
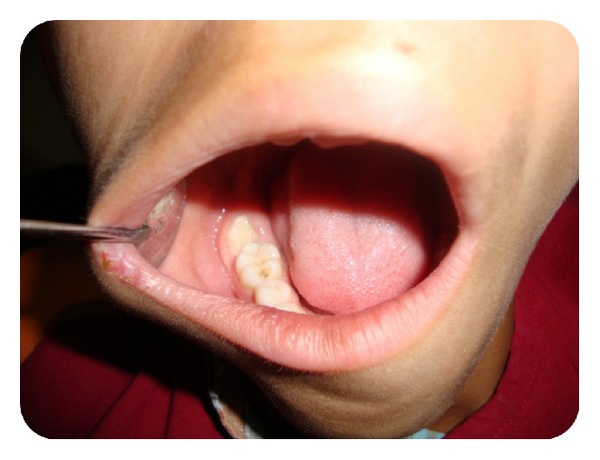
Intraoral operative photograph showing second molar partly covered with pericoronal flap partly.

**Figure 2:**
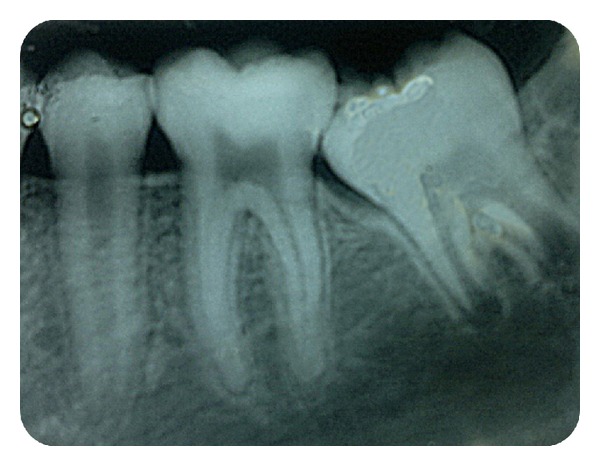
Intraoral periapical radiograph showing permanent mandibular second molar locked beneath first permanent molar.

Clinically, the right mandibular second molar was partly visible (Figure 1). There was no tooth mobility and no tenderness to percussion in relation to the right mandibular first permanent molar. An intraoral periapical radiograph was advised. Radiograph revealed a mesially inclined right mandibular second molar which was partly underneath the cement-enamel junction of the adjacent tooth and partly above it. The roots of the second mandibular molar were immature with an open apex (Figure 2) and no resorption facets were detected on the first molar. Correlating clinical and radiographic findings, the right mandibular second molar was diagnosed as ectopic.

**Figure 3:**
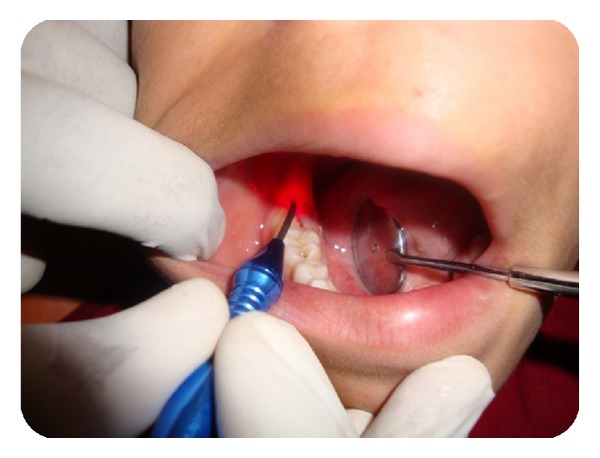
Excision of the pericoronal flap with diode laser.

**Figure 4:**
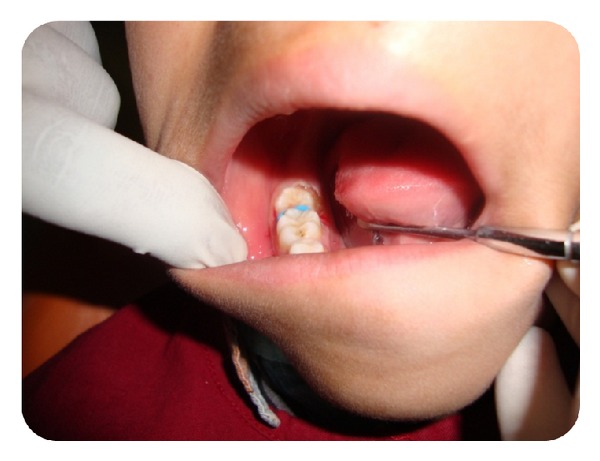
Elastic separator placed interproximally.

**Figure 5:**
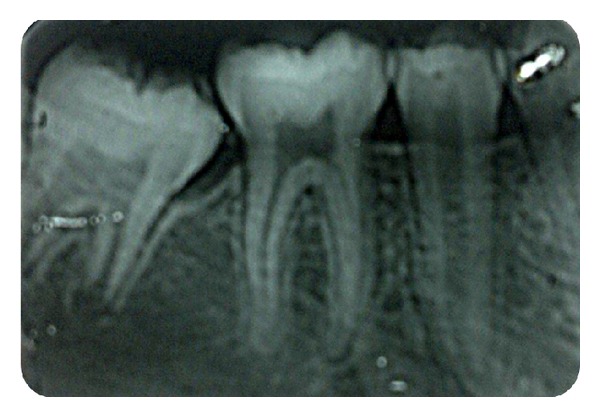
Intraoral periapical radiograph after 3 days with elastic placed.

The first step in treatment is to expose the permanent second molar completely and to have better access. Under topical anaesthesia the mucosa or the pericoronal flap overlying the second molar was excised, using a diode laser (Figure 3). In order to place the elastic separators in the same appointment, laser was preferred over surgical excision, as it provides better visibility and a blood free field. The second goal of the treatment was to place an elastic separator. The elastic separator acts as a wedge and aids in pushing the molar distally to gain space. Initially a single Dynaflex posterior blue elastic separator with a diameter of 2.5 mm was stretched and placed. Patient was called for review after 3 days (Figure 4). Mild distal movement of the molar was observed (Figure 5).

**Figure 6:**
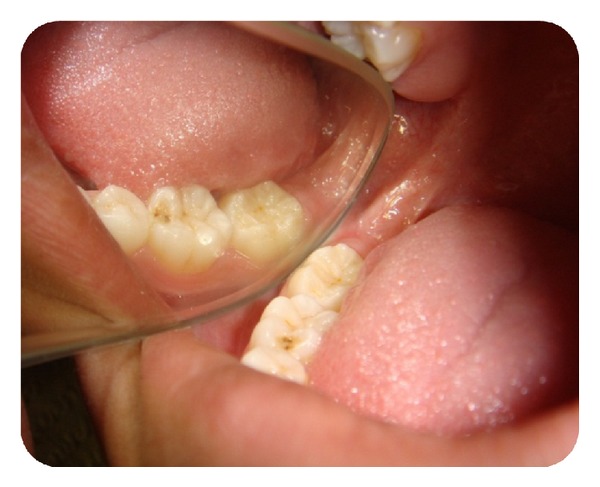
Posttreatment photograph with permanent mandibular second molar erupted.

**Figure 7:**
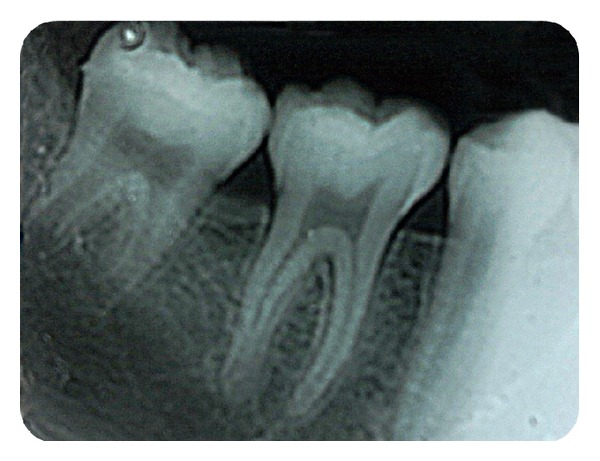
Posttreatment intraoral periapical radiograph showing erupted permanent mandibular second molar erupted.

One more separator was added to overcorrect it and the patient was asked to revisit in 3 days. On the second recall visit, elastics were removed as the tooth was upright and free to erupt (Figures 6 and 7). No further treatment was advised as the tooth was expected to move into occlusion with a normal eruptive force. After 1-month followup, the patient was found to be asymptomatic.

## 3. Discussion 

Ectopic eruption of second molar is relatively rare. In the earlier reports titanium screw implants, titanium molybdenum alloy tip-back cantilever, Ni-Ti coils, and few others were used along with surgical exposure [9, 10]. The concept of space gaining by method of interproximal wedging through the use of separators was restricted to first permanent molar but none have reported the use of elastic separators in treating ectopic second permanent molar. The choice of this treatment was based on the assumption that there is eruptive force remaining in the molar which can guide the tooth to erupt once the tooth overcomes obstruction. Other traditional treatment modalities for space regaining by interproximal wedging are brass wire, metal separators, and deimpactor spring [11–13]. Since there was limited access to interproximal region and discomfort brass wire, metal separators and deimpactor spring were not used.

The traditional treatment modalities have several disadvantages over the present protocol.

Gingival inflammation around the screws, poor oral hygiene, inapplicability in poor bone support areas, and treatment costs are the main disadvantages. The advantages of using elastic separators are ease of use and placement, short span of force application, unnecessary band or bonding of adjacent teeth, and cost effectiveness. Disadvantage of this procedure is that it may result in unwanted movement of the tooth.

In conclusion, we would like to advocate the use of simple procedures and cost effective treatment modalities for better comfort and satisfaction of the patient. Hence, dentists should be aware of the basic procedures along with recent advances.
